# Ammonia level at admission predicts in-hospital mortality for patients with alcoholic hepatitis

**DOI:** 10.1093/gastro/gow010

**Published:** 2016-05-01

**Authors:** Sujan Ravi, Kaely S. Bade, Mohsen Hasanin, Ashwani K. Singal

**Affiliations:** 1 Department of Internal Medicine, University of Alabama at Birmingham, Birmingham, AL, USA; 2 Division of Gastroenterology and Hepatology, University of Missouri, Columbia, MO, USA; 3 Division of Gastroenterology and Hepatology, University of Alabama at Birmingham, Birmingham, AL, USA

**Keywords:** alcoholic hepatitis, hepatic encephalopathy, ammonia, mortality

## Abstract

**Objective.** Alcoholic hepatitis (AH), a unique clinical syndrome among patients with chronic and active alcohol use, is associated with high short-term mortality. An elevated ammonia level is associated with mortality in patients with acute liver failure; however, its impact in AH has not been well-studied.

**Methods.** A retrospective study was performed on patients admitted to a tertiary-care hospital with the discharge diagnosis of AH. Patients meeting criteria for AH were included in the final data analysis. Multivariate logistic regression models were built to examine the impact of serum ammonia in predicting in-hospital mortality (IHM) and 30-day mortality (TDM). Subgroup analysis was also performed, which was limited to patients who had hepatic encephalopathy.

**Results.** Of the 105 AH patients included, 26 (25%) died during the initial hospitalization. Among the 79 patients who survived initial hospitalization, 30 (39%) died within 30 days. Information about ammonia levels at admission was available for 82 patients. Of these, 25 patients had IHM and significantly higher ammonia level (97 *vs.* 69 μmol/L, *P* < 0.01). Among the 57 who survived hospitalization, ammonia levels were not significantly different (71 *vs.* 67 μmol/L, *P* = 0.69) in patients with and without TDM. The addition of ammonia to the multivariate regression models including age, sex, cirrhosis, treatment and model for end-stage liver disease (MELD) score improved the C statistics for IHM from 0.708 to 0.801 and for TDM from 0.756 to 0.766, respectively. These results were identical, even when limited to patients with hepatic encephalopathy.

**Conclusion**. AH patients with elevated ammonia levels at admission have higher IHM; however, they do not seem to play a significant role in 30-day mortality for patients who survived hospitalization.

## 
INTRODUCTION

Alcoholic hepatitis (AH) is a syndrome of liver failure and jaundice in patients with heavy and long-standing alcohol consumption [1]. Hepatic encephalopathy (HE) is a clinical manifestation of severe AH and has been associated with high mortality [2]. Furthermore, pharmacological options for the treatment of severe alcoholic hepatitis are limited. The recently published STOPAH study ruled out the benefits of pentoxifylline and seriously questioned the benefit of corticosteroids [3]. The pathogenesis of HE is multifactorial, but elevated serum ammonia plays a pivotal role [4,5]. In patients with liver failure, impaired hepatic clearance is a major contributor to hyperammonemia in addition to other mechanisms including metabolic and biochemical pathways in the kidneys and muscles [4]. The current evidence evaluating ammonia levels and its relationship with the severity of HE is conflicting [6–8]. Studies evaluating the prognostic implication of ammonia levels by itself are limited. Among patients with acutely decompensated cirrhosis, an elevated ammonia level at admission was individually associated with higher 30- and 90-day mortality [9]. In hospitalized AH patients in the United States, hyperammonemia within 2 days of admission was associated with higher 28-day mortality [10]. There are several prognostic models to risk-stratify patients with AH [11]. Among the available prognostic scores, only the Child-Turcotte-Pugh score uses HE in its model, and none of the current models include ammonia levels for risk stratification. We performed this study to evaluate the impact of ammonia levels on in-hospital mortality (IHM) and 30-day mortality (TDM).

## METHODS

### Study design and population

After obtaining permission from the institutional review board at the University of Alabama at Birmingham (UAB), a retrospective chart review was performed on patients discharged between January 2005 and December 2013 with the diagnosis of AH (ICD-9 code 571.1). AH was defined using the following criteria: (i) alcohol use of > 50 g/d in men and >30 g/d in women for > 5 years, (ii) last drink within three weeks of admission, (iii) serum bilirubin > 5 mg/dL, (iv) elevated transaminases but not above 400 IU/L, (v) aspartate transaminase (AST) to alanine transaminase (ALT) ratio > 2:1 and (vi) exclusion of other concomitant liver disease. For patients with > one admission, information was collected only for the first admission.

### Data collection

Two authors (SR and KB) independently performed chart review to confirm the AH diagnosis and collect data on (i) demographics (age, sex and ethnicity), (ii) dates of admission, discharge, last follow up and death, (iii) laboratory data: AST, ALT, white blood cell (WBC) count, prothrombin time (PT), international normalized ratio (INR), bilirubin, creatinine, blood urea nitrogen (BUN), albumin and ammonia at admission. Ammonia levels were performed similarly across the study population and collected from venous samples, (iv) clinical or radiological evidence of cirrhosis and complications including HE, (v) treatment with steroids and/or pentoxyfylline and (vi) study outcomes on IHM and TDM. The available information was used to calculate Maddrey’s discriminant function (MDF), model for end-stage liver disease (MELD) score and Glasgow alcoholic hepatitis score (GAHS). Mortality data were also verified with the U.S. Social Security database.

### Statistical analysis

Statistical analysis software (SAS Institute, Cary, North Carolina) version 9.3 was used for our statistical analysis. Baseline characteristics of patients with and without IHM and TDM were compared using chi-square and Sstudent’s t tests for categorical and continuous variables, respectively. Factors that were clinically relevant and/or had statistically significant differences (*P* < 0.05) in patients with and without IHM and TDM were used to build multivariate logistic regression models. Ammonia level was then added to the model to evaluate for improvement in the model design. Receiver operator characteristic (ROC) curves were also used to evaluate the impact of ammonia. *P* values < 0.05 were considered to be statistically significant. Subgroup analysis was also performed, limited to patients with HE.

## RESULTS

### Study population

**Figure 1.**
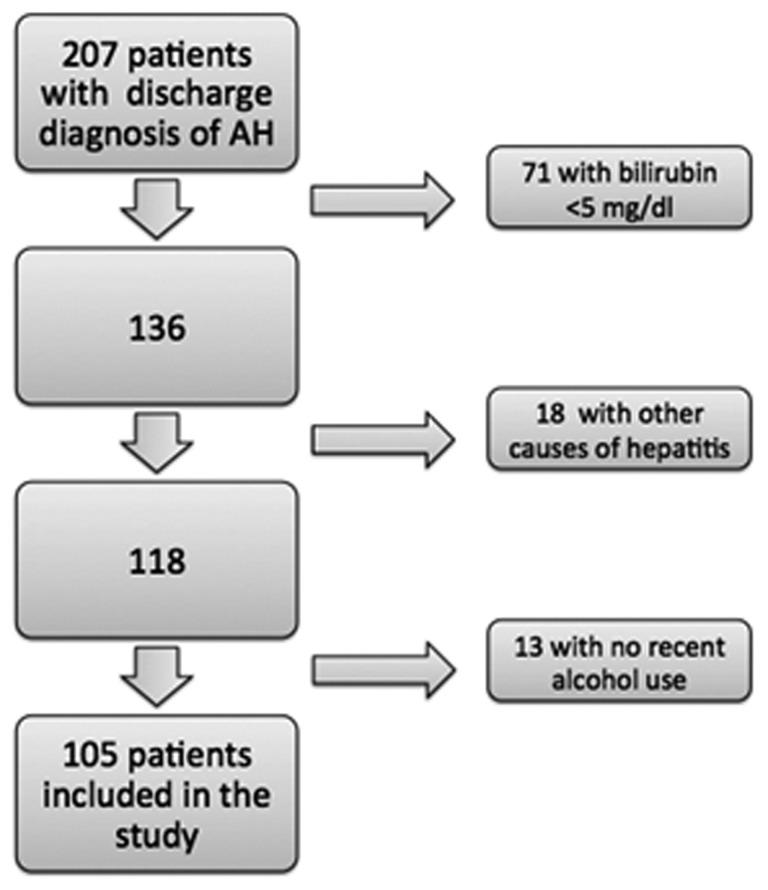
Study attrition and patient selection.

A total of 207 patients were admitted to our center between December 2004 and December 2012 with a discharge diagnosis of AH. After excluding patients who did not meet the criteria for AH (Figure 1**),** 105 patients were included in the study (mean age 48 years, 66% males, 81% Caucasians, 56% cirrhosis and mean MDF, MELD and GAHS scores of 54, 25 and 8, respectively). A total of 79% of the patients had a severe episode of alcoholic hepatitis (MDF ≥ 32), and 50% were treated (39 patients received corticosteroids, and 13 received pentoxyfylline).

### In-hospital and 30-day mortality

A total of 26 patients (25%) patients died during hospitalization (IHM) with mean length of hospital stay of about 15 days. Among the 79 patients discharged alive from the hospital, 30 (39%) died within 30 days of the initial hospitalization (TDM).

### Baseline characteristics

Patients with IHM compared with those discharged alive were different for higher frequency of cirrhosis (64% *vs.* 38%, *P* = 0.02) and HE (100% *vs.* 32%, *P* < 0.01) as well as higher WBC count (16.2 *vs.* 11.8 ×10^6^/L, *P* = 0.02). They also had worse liver function with higher serum bilirubin (21 *vs.* 14 mg/dL, *P* = 0.02), MDF (68 *vs.* 50, *P* = 0.01), MELD (30 *vs.* 24, *P < *0.01) and GAHS (8.9 *vs.* 7.7, *P*<0.01). Among 79 survivors of initial hospitalization, patients dying within 30 days after initial hospitalization compared with those surviving beyond 30 days had higher bilirubin (18 *vs.* 12 mg/dL, *P* = 0.04), creatinine (2.2 *vs.* 1.3 mg/dL, *P*= 0.04), MDF (60 *vs.* 43, *P* = 0.03) and GAHS (8.3 *vs.* 7.3, *P* < 0.01) (Table 1).

**Table 1.** Baseline characteristics of patients with alcoholic hepatitis: comparing patients with and without in-hospital mortality and 30-day mortality among survivors of initial hospitalization

Characteristics	No IHM (*n* = 79)	IHM (*n* = 26)	*P* value	No TDM (*n* = 49)	TDM (*n* = 30)	*P* value
Age (years, mean ± SD)	47 ± 11	51 ± 10	0.07	47 ± 11	47 ± 10	0.84
Sex (% male)	38	77	0.17	63	60	0.83
Race (% Caucasians)	76	81	0.64	75	77	0.24
Cirrhosis (%)	38	64	0.02	42	33	0.46
Hepatic encephalopathy (%)	32	100	<0.01	27	40	0.23
WBC count (×10^6^/L, mean ± SD)	11.8 ± 7.9	16.2 ± 9.8	0.02	11.6 ± 8.7	14.3 ± 8.4	0.11
AST (IU/L, mean ± SD)	198 ± 107	233 ± 180	0.23	210 ± 109	178 ± 104	0.21
ALT (IU/L, mean ± SD)	73 ± 47	81 ± 58	0.5	81 ± 50	62 ± 41	0.09
Bilirubin (mg/dL, mean ± SD)	14 ± 11	21 ± 13	0.02	12 ± 10	18 ± 13	0.04
Creatinine (mg/dL, mean ± SD)	1.6 ± 1.8	2.3 ± 1.9	0.12	1.3 ± 0.9	2.2 ± 2.5	0.03
BUN (mg/dL, mean ± SD)	22 ± 25	27 ± 24	0.4	18 ± 21	29 ± 31	0.07
Albumin (g/dL, mean ± SD)	2.5 ± 0.7	3.5 ± 6.9	0.19	2.5 ± 0.7	2.5 ± 0.7	0.9
MDF score (mean ± SD)	50 ± 33	68 ± 27	0.01	43 ± 30	60 ± 36	0.03
MELD score (mean ± SD)	24 ± 10	30 ± 8	<0.01	22 ± 8	27 ± 12	0.05
GAHS (mean ± SD)	7.7 ± 1.7	8.9 ± 1.4	<0.01	7.3 ± 1.6	8.3 ± 1.8	<0.01
Treatment (%)	48	54	0.61	44	57	0.27

SD, standard deviation

### Admission ammonia level and mortality

Information about ammonia levels at admission was available for 82 patients. Patients with (*n*=82) and without (*n*=23) available ammonia levels were similar in their demographic characteristics and laboratory parameters including WBC count, albumin, AST, ALT and creatinine. Patients with available ammonia levels had a higher proportion with encephalopathy (57% *vs.* 17%, *P* = 0.007)*.* These patients also had higher mean MELD (27 *vs.* 20, *P* = 0.004), mean GAHS (8 *vs.* 7, *P* = 0.001) and mean MDF scores (59 *vs.* 36, *P* = 0.003).

**Figure 2.**
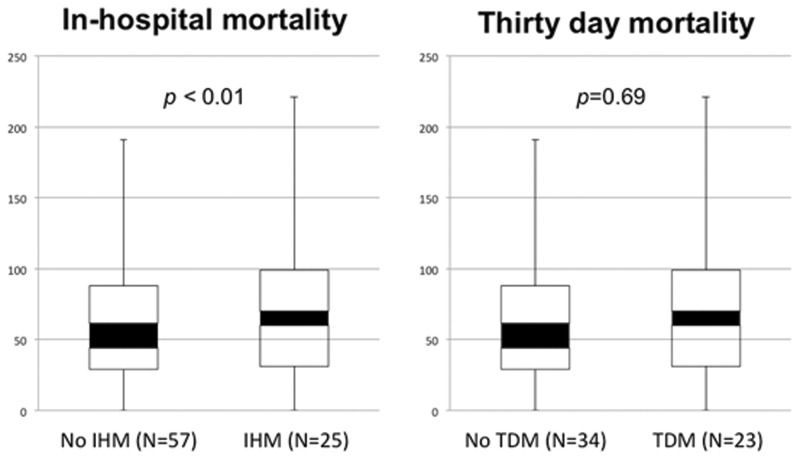
Serum ammonia level at admission comparing patients with and without in-hospital mortality and patients with and without mortality at 30 days.

**Figure 3.**
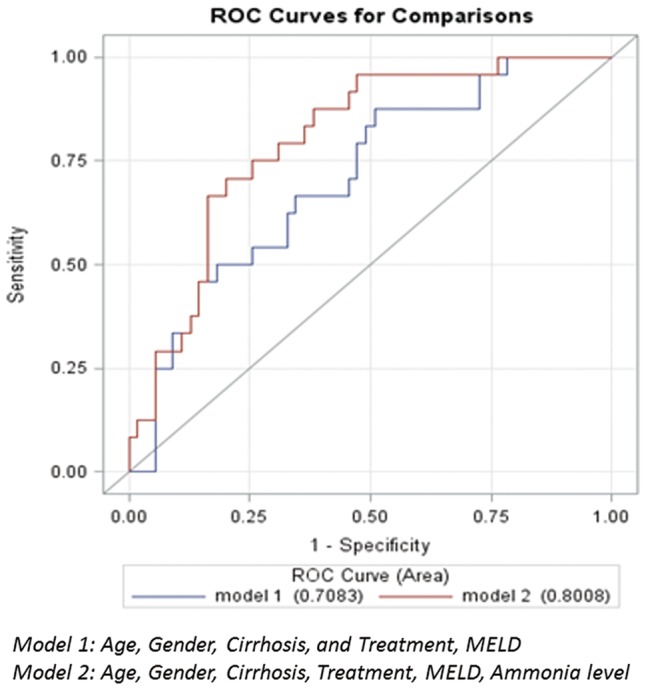
Receiver operating characteristic **(**ROC) curves on prediction of in-hospital mortality among hospitalized alcoholic hepatitis patients.

Among the 82 patients with available ammonia levels, 25 patients with IHM compared with 57 without IHM had higher ammonia levels (97 *vs.* 69 μmol/L, *P* < 0.01); however, in the 57 patients who survived hospitalization, ammonia levels were not different when comparing patients with and without TDM (71 *vs.* 67 μmol/L, *P* = 0.69) (Figure 2). Multivariate regression models including age, sex, cirrhosis, treatment and MELD score were built to evaluate predictors for IHM and TDM. C statistics for these models were 0.708 and 0.756, respectively. Admission ammonia level added to the model independently predicted IHM, which improved the C statistic to 0.801 (Table 2) and improved the area under the curve (AUC) (Figure 3**);** however, admission ammonia level did not predict TDM, and its addition to the model only improved the C statistic to 0.766 (Table 2).

**Table 2.** Cox proportional hazard regression model to analyze factors predicting in-hospital mortality and 30-day mortality among hospitalized alcoholic hepatitis patients

Variables	IHM				TDM			
	Model 1		Model 2		Model 1		Model 2	
	OR	95% CI	OR	95% CI	OR	95% CI	OR	95% CI
Age	1.05	0.99–1.11	1.05	0.99–1.12	1.08	1.02–1.14	1.05	0.98–1.12
Sex	0.72	0.22–2.37	0.48	0.12–1.89	1.67	0.53–5.19	1.09	0.3–4.0
Cirrhosis	1.80	0.62–5.23	1.71	0.54–5.42	0.54	0.19–1.58	0.23	0.06–0.96
Treatment	1.39	0.48–4.03	1.47	0.47–4.59	1.55	0.53–4.51	0.65	0.44–0.52
MELD	1.04	0.99–1.10	1.03	0.97–1.09	1.08	1.02–1.14	1.08	1.01–1.16
Ammonia	-		1.03	1.01–1.04	-		0.996	0.97–1.02
	C = 0.708		C = 0.801		C = 0.756		C = 0.766	

CI, confidence interval; OR, odds ratio

### Subgroup analysis of patients with hepatic encephalopathy

**Figure 4.**
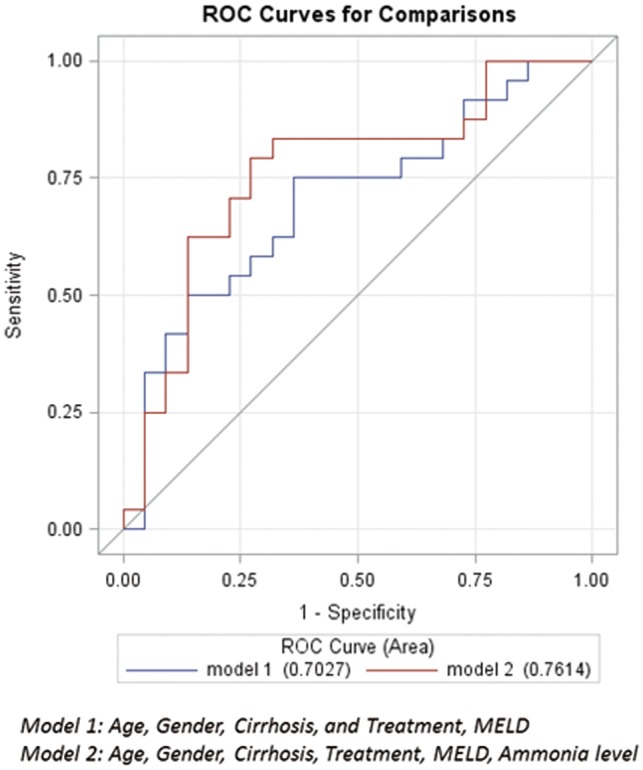
ROC curves on prediction of in-hospital mortality among hospitalized alcoholic hepatitis patients with hepatic encephalopathy.

Of the study population, 51 patients had HE at or during the hospitalization, with admission ammonia levels being available for 47 patients. Also within this group, 25 patients with IHM tended to have a higher ammonia level at admission compared with the 26 patients who survived the hospitalization (96 *vs.* 75 μmol/L, *P=* 0.08). Admission ammonia level added to a multivariate regression model with age, sex, cirrhosis, treatment and MELD improved the C statistic from 0.703 to 0.761 (Table 3) and improved the AUC (Figure 4).

**Table 3.** Cox proportional hazard regression model to analyze factors predicting in-hospital mortality among hospitalized alcoholic hepatitis patients with hepatic encephalopathy

Variables	IHM			
	Model 1		Model 2	
	OR	95% CI	OR	95% CI
Age	1.07	0.995–1.14	1.07	0.995–1.16
Sex	2.27	0.41–12.64	1.19	0.18–7.73
Cirrhosis	1.12	0.30–4.27	1.20	0.30–4.84
Treatment	1.05	0.29–3.88	1.16	0.30–4.47
MELD	1.02	0.96–1.09	1.02	0.95–1.08
Ammonia	-		1.02	0.997–1.04
	C = 0.703		C = 0.761	

CI, confidence interval; OR: odds ratio

## DISCUSSION

Our study shows that, among hospitalized patients with AH, elevated admission ammonia level is associated with IHM. Although HE is the strongest clinical factor associated with IHM, the admission ammonia level improves the prediction of mortality during hospitalization even in patients with HE. The admission ammonia level was not associated with survival at 30 days, once the patient had survived the initial hospitalization.

In one retrospective study of patients with decompensated cirrhosis, mortality at 30 and 90 days was reported at 23% and 38%, respectively. In that study, the admission ammonia level was independently associated with death or transplant at 30 and 90 days [9]. TDM assessed in our study is not comparable with that study as we excluded patients who died during their hospital stay. In another study, hyperammonemia within the first two days of admission independently predicted 28-day mortality in hospitalized AH patients in the United States. However, this association did not hold valid after adjusting for the peak MELD score during hospitalization. Differences in analytic methods could explain these variable results as ammonia level was used as a continuous variable in our study and not as a categorical variable as used in the study by Patwardhn *et al*. [9] Hyperammonemia was also not significantly predictive of 90-day mortality in the study by Cuthbert *et al.*, similar to data from our study [10]. 

To the best of our knowledge, our’s is the first study to report the impact of serum ammonia levels on IHM in hospitalized AH patients. We believe the completeness in data collection and strict case definition contribute to the strength of our study; however, our study is limited by a retrospective study design with selection of patient population based on medical coding. However, we carefully reviewed all of the charts to characterize the patient population. More severe disease among patients with available ammonia levels compared with patients with unavailable ammonia levels is a potential bias and another limitation of this retrospective study. Further larger prospective studies are suggested to validate this finding as a basis to incorporate admission ammonia level into the prognostication for hospitalized AH patients.

In conclusion, AH patients with elevated ammonia levels at admission have higher in-hospital mortality; however, they do not appear to play a significant role in 30-day mortality of patients who have survived hospitalization.

**Conflict of interest statement:** none declared.
